# Birth preparedness and complication readiness practice and associated factors among pregnant women in Northwest Ethiopia: 2018

**DOI:** 10.1371/journal.pone.0249083

**Published:** 2021-04-22

**Authors:** Tibeb Zena Debelie, Abdella Amano Abdo, Kiber Temesgen Anteneh, Miteku Andualem Limenih, Mengstu Melkamu Asaye, Getie Lake Aynalem, Worku Mequannt Ambaw, Belayneh Ayanaw Kassie, Solomon Mekonnen Abebe

**Affiliations:** 1 Department of Clinical Midwifery, School of Midwifery, College of Medicine and Health Sciences, University of Gondar, Gondar, Ethiopia; 2 College of Medicine and Health Sciences, Hawassa University, Hawassa, Ethiopia; 3 Department of Women’s and Family Health, School of Midwifery, College of Medicine and Health Sciences, University of Gondar, Gondar, Ethiopia; 4 Institute of Public Health, College of Medicine and Health Sciences, University of Gondar, Gondar, Ethiopia; 1. IRCCS Neuromed 2. Doctors with Africa CUAMM, ITALY

## Abstract

**Background:**

Birth-preparedness and complication readiness is a comprehensive strategy aimed at promoting the timely utilization of skilled maternal and neonatal health care. Pregnancy-related complications both on the mother and the newborn could be largely alleviated if there is a well-consolidated birth preparedness and complication readiness plan developed during pregnancy and implemented at the time of delivery.

**Objective:**

To determine the prevalence of birth preparedness and complication readiness practice (BPCR) and associated factors among pregnant women in North Gondar Zone, Northwest Ethiopia, 2018.

**Methods:**

A community based cross-sectional study was conducted among pregnant women in North Gondar Zone from March 2017 to February 2018. A multistage clustered sampling technique was used to enroll a total of 1620 participants. The data were collected by face to face interviews using pretested and semi-structured questionnaires at baseline and following delivery. The data were entered using EPI-data version 3.1 and analyzed using STATA version 14 software. Bivariate and multivariable logistic regression model was fitted to assess factors with BPCR practice. Adjusted odds ratio (AOR) with 95% confidence interval was used to determine the association between covariates and the outcome variable.

**Results:**

From a total of 1620 pregnant women only 1523 (94.0%) mothers were followed at the end line. The prevalence of BPCR plan during pregnancy was 66.1% [95% CI: 63.8, 68.5] and the practice at the time of delivery was 73.5% [95% CI 71.3, 75.7]. Of the total respondents who mentioned having a BPCR plan, 76.4% practiced at the time of delivery. Frequency of ANC visits [AOR = 1.97; 95% CI: 1.67, 2.32], larger number of family in the household [AOR = 1.14; 95%CI: 1.00, 1.30], highest wealth asset [AOR = 1.87; 95%CI: 1.16, 3.01], Multigravidity [AOR = 0.30; 95% CI: 0.15, 0.62], husband involvement in decision making [AOR = 2.2; 95% CI: 1.25, 3.82], counseled on BPCR [AOR = 2.35; 95% CI: 1.51, 3.68], were found to be significantly associated with BPCR practice.

**Conclusion:**

BPCR practice at the time of delivery was higher than previous studies conducted in the country. However, BPCR practice was found to be lower than the standard that every woman should practice the plan at the time of delivery. Intersectoral collaborative interventions required to improve the economic status and living standard of families in the community as well as various awareness creation strategies should be implemented to support women to attend ANC follow-up visits.

## Introduction

Birth-preparedness and complication readiness is a comprehensive strategy aimed at promoting the timely utilization of skilled maternal and neonatal health care. It is a programming tool to address delay causing factors (delay in seeking care, delay in reaching care, and delay in receiving care)at various levels [1,2]. It is one of the essential interventions that WHO recommended being included in the ANC services package [3].

Pregnancy and childbirth-related problems are major public health challenges for many countries in the world. In Sub-Saharan Africa, in the year 2017, there was a total of 542 maternal deaths per 100,000 live births which are nearly 2/3 of the total maternal deaths worldwide. Ethiopia is one of the 15 countries in the world which is considered to be high alert areas. Also, among the countries that contribute to the high maternal mortality ratio (MMR) [4,5].

Women die as a result of complications that arise during pregnancy, childbirth, and the postpartum period. Other complications may exist before pregnancy but are worsened during pregnancy [6]. The major complications that account for 80% of all maternal deaths are severe bleeding (mostly bleeding after childbirth), infections (usually after childbirth), high blood pressure during pregnancy (preeclampsia and eclampsia), unsafe abortion [7,8]. Most maternal deaths are avoidable, as the health-care solutions to prevent or manage complications are well known [9]. Those problems can be alleviated by implementing a basic Maternal and Child Health (MCH) service [10]. All women need access to high-quality care provided by skilled health professionals during pregnancy, during childbirth, and care and support in the weeks after childbirth [11]. Study findings showed births attended by skilled health care professionals improves the chance of survival both for the mother and the fetus [12,13].

Preparing the women during pregnancy for labor and delivery and possible complications that may arise at any stage of the process will play a major role in decreasing maternal mortality and morbidity [14]. Engaging holistic stakeholders including women, her families, the community, facilities, and providers and preparing for labor and the possible complications will help to avoid the delays in decision making to seek care, accessing service, and getting care in facilities [15]. ANC follow up is one of the interventions that keep the course of the pregnancy safer and to prepare the women to deliver at health facilities where she gets high-quality service.

The elements of BPCR are deciding the place of delivery, knowing the preferred birth attendant, saving money, identifying compatible blood donor and labor companion, choosing support person to look after the home and other children, arranging necessary supplies and materials for labor and delivery, preparing transportation services [16]. Survey data from Ethiopia other areas indicate that there is a scarcity of combined research on women’s BPCR plan during pregnancy and actual practice at the time of delivery. Therefore, this study aimed to assess the magnitude of birth preparedness and complication readiness plan during their pregnancy and practice during childbirth and its associated factors using a prospective cohort follow-up study design in Northwest Ethiopia.

## Methods

### Study design, setting and period

A community-based cross-sectional study was conducted among pregnant woman in North Gondar Zone, Ethiopia, from March 2017 to February 2018. North Gondar is found in Amhara regional state, it recently reorganized in to three administrative zones (North Gondar, Central Gondar, and West Gondar). It has an estimated population of 1,477,931. There are a total of 138 health centers and 15 hospitals providing services for the community.

### Study population

All pregnant women who are permanently residing (at least for the last 6 months) in the selected urban and rural Kebeles of North Gondar Zone were included in the study. Pregnant women who were severely ill and unable to respond were excluded from the study. The study followed all pregnant women from pregnancy up to 6 weeks post-partum.

### Study size

The sample size was determined using a single population proportion formula considering the following statistical assumptions: Magnitude of post-partum family planning was 12.3% taken from a study done on postpartum family planning utilization in Kebribeyah town, Ethiopia [17]. Level of significance 5%, Z α/2 = 1.96, and Absolute precision or margin of error = 2%, the calculated sample size is 1035. With a design effect of 1.5 and loss to follow up 5%, the final sample size was 1631.

### Sampling technique

A Multistage clustered sampling technique was used to select the woredas in North Gondar Zone. Initially, 20% (5) of the Woredas were selected using a simple random sampling technique. Then 58 Kebeles were selected from a total of 148 kebeles in the five selected Woredas using a simple random sampling technique. All pregnant mothers in the selected Kebeles were the sampling units at the baseline. In case where the samples were not adequate in one Kebele, the nearby Kebeles were included.

### Data collection tools and procedure

Data were collected by face to face interview using a semi-structured questioner adapted from the safe motherhood questionnaire developed by maternal and neonatal health program of the Johns Hopkins Program For International Education In Gynecology and Obstetrics (JHPIEGO), to determine BPCR plan and practice of the women [15].

The dependent variables were birth preparedness and complication readiness practice. Independent variable like sociodemographic characteristics (age, ethnicity, marital status of the mother, educational status of the mother, occupation, husband’s educational status, husband’s occupational status, residence, household wealth index, decision making on maternal health care service utilization) were collected. Obstetric characteristics **(**Number of living children, number of pregnancy, the outcome of last pregnancy, outcome of the last birth, Mode of delivery of last birth, pregnancy complications, frequency of ANC visit, and BPCR plan) were taken. Data collection of BPCR plan was done at the baseline, and women were followed up to delivery to collect actual BPCR practice following delivery.

Five criteria were set to determine BPCR these are identifying the place of delivery, choosing skilled birth attendants, saving money for labor and delivery, arranging transportation, and blood donor. Women who said they had planned three of the five components were considered as having a BPCR plan during pregnancy. During delivery, if they had at least three of the five components at hand, they were considered practicing BPCR. Prepared mothers were given 1 while non-prepared mothers were given 0. The cutoff point was implemented by various works of literature in the past [18–20].

### Data quality assurance

To ensure data quality, questioner first prepared in English and translated back to Amharic and back to English to maintain consistency. Thirty data collectors were recruited and ten supervisors monitored the data collection process. Three days of training was provided both for data collectors and supervisors. The data collection tool was pretested among pregnant women out of the study area on 5% of the total sample size. The data collection process was closely supervised and the collected data were checked daily for completeness and consistency.

### Data processing and analysis

The data was cleaned and coded manually to ascertain completeness and consistency. Then it was entered into EPI data version 3.1 and exported to STATA version 14 for analysis. Descriptive statistics were performed and explained with tables and graphs. Wealth index was analyzed using principal component analysis. Logistic regression was used to identify factors associated with BPCR practice. Variables with a p-value of 0.2 in bivariate analysis were entered into a multivariable logic regression model and considered statistically significant at a P-value of <0.05. The adjusted odds ratio with a 95% CI interval was used to determine the degree and direction of the association between covariates and the outcome variable.

### Ethical approval and consent to participate

Ethical clearance was obtained from the Ethical Review Board of the University of Gondar with Ref. No.O/V/P/RCS/05/310/2017. Written consent was obtained from each study participant after informing the objective of the study. In the consent, statements about the potential risk, benefit, and confidentiality were included. Participants were informed that they had the right to withdraw from the study at any time and also, informed written consent was obtained from participants before conducting the interview.

## Results

### Socio-demographic characteristics

In the selected Kebeles there were a total of 1625 pregnant women at the time of data collection. Out of these 5 individuals were not willing to participate in the study. We enrolled a total of 1620 pregnant mothers to the cohort at the baseline (during pregnancy). At the end line (during delivery), there were a total of 1523 participants involved in the data collection making a loss to follow up rate of 5.98%. The mean age of the respondents was 27.7 ±5.78 years. Regarding marital status 1461 (95.93%) of the respondents were married. Six hundred seventy-six (44.39%) of the mothers were not able to read or write. The majority of the respondents (93.96%) belong to Amhara ethnicity. Among the respondents, 1000(65.7%) were housewives. Nearly a third of the participants stated they had no access to public transportation (27.3%) **(Table 1)**.

**Table 1:** Socio-demographic characteristic of study participants at North Gondar Zone, 2018 (n = 1523).

Variables	Frequency (%)
**Religion**	
Orthodox	1,307(85.82)
Muslim	210(13.79)
Others*	6(0.39)
**Ethnicity**	
Amhara	1,431(93.96)
Qimant	62(4.07)
Others**	30(1.97)
**Mothers education**	
unable to read and write	676(44.39)
able to read and write	158(10.37)
primary education	252(16.55)
secondary education	259(17.01)
college and above	178(11.69)
**Mothers Occupation**	
Housewife	1,000(65.66)
Farming	223(14.64)
Self-Employee	129(8.47)
Government Employee	127(8.34)
Others***	44(2.89)
**Husbands education (N = 1486)**	
unable to read and write	470(31.63)
able to read and write	319(21.47)
primary education	249(16.76)
secondary education	251(16.89)
college and above	197(13.26)
**Husbands occupation**	
Farming	793(52.07)
Government Employee	199(13.07)
Daily Laborer	81(5.32)
Self-Employee	349(23.52)
Others****	62(4.18)
**Residence**	
Rural	692(45.44)
Urban	831(54.56)
**Wealth index**	
Lowest	306(20.9)
Second	317(20.81)
Middle	302(19.83)
Fourth	301(19.76)
Highest	297(19.50)

*Catholic, Protestant

** Oromo, Tigre

*** Student, Daily laborer

****Private employee, Student.

### Obstetric characteristics of the respondents

Among the study participants, 435 (28.56%) reported this was their first pregnancy. Most participants 1371(90%) and 1487(97.6%) reported their pregnancy was planned and wanted respectively. From the participants who had previous pregnancy (child birth experiences), 154(14.37%) had 5 or more alive children. The majority of the respondents (67.97%) reported they started their ANC follow up after the first trimester (16 weeks of gestation). Among the multiparous respondents, (6.14%) had a history of stillbirth whereas, 133 (12.2%) had pregnancy-related complications in the past. Six hundred one (55.6%) of the respondents stated they had institutional delivery in the past **(Table 2)**.

**Table 2:** Obstetric characteristics of study participants at North Gondar Zone, 2018 (n = 1523).

Variable	Frequency (%)
**Number of pregnancies**	
1	435(28.56%)
2–4	740(48.59%)
> = 5	348(22.85%)
**Number of alive children (N = 1072)**	
0–1	325(30.32)
2–4	593(55.32)
> = 5	154(14.37)
**Places of last childbirth (N = 1081)**	
Home	480 (44.4)
Health facility	601 (55.6)
**Mode of delivery of last birth (N = 1081)**	
SVD	990 (91.58)
C/S	47 (4.35)
Instrumental	44 (4.07)
**Health problems faced during the last pregnancy (N = 1084)**	
No	911 (84.04)
Yes	173 (15.96)
**The outcome of last pregnancy (N = 1080)**	
Alive	1020 (93.75)
Stillbirth	30 (2.76)
Abortion	38 (3.49)
**Decision making on maternal health care utilization**	
Me alone	101 (6.63)
Me and my husband	1348 (88.51)
Others	74 (4.85)
**Knowledge of birth preparedness and complication readiness**	
No	948 (62.25)
Yes	575 (37.75)
**Knowledge of danger signs of pregnancy**	
No	1001 (65.73)
Yes	522 (34.27)
**Had ANC for the current pregnancy**	
No	111 (7.29)
Yes	1412 (92.71)
**Time of ANC initiation in Months**	
Early (>4 months)	452 (32.03)
Late (> = 4 months)	959(67.97)
**Presence of danger signs for the current pregnancy**	
No	1392 (91.4)
Yes	131 (8.6)
**Counseled about birth preparedness and complication readiness plan**	
No	1247 (81.88)
Yes	276 (18.12)
**Counseled about institutional delivery**	
No	799 (52.46)
Yes	724 (47.54)

### Prevalence of BPCR

**Fig 1:**
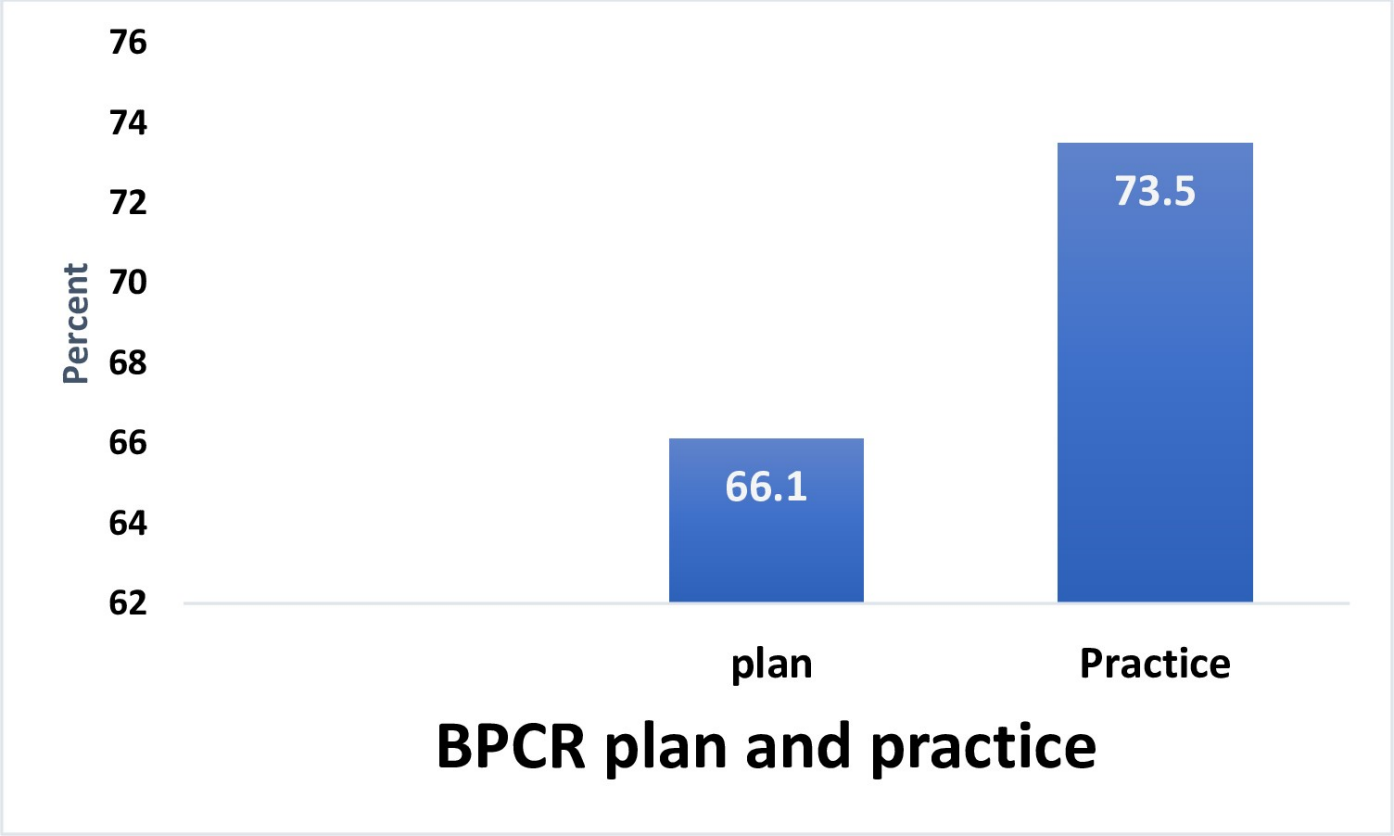
BPCR plan and practice among the cohort of pregnant women in North Gondar Zone, Northwest Ethiopia, 2018.

The prevalence of the BPCR plan of the women was estimated to be 66.1 [95% CI (63.8,68.5)] during her pregnancy and the actual practice at the time of birth increased to 73.5 [95% CI 71.3–75.7]. From the respondents that said they had a BPCR plan during pregnancy, 23.6% failed to practice it at the time of delivery (**Fig 1**).

### BPCR plan and practice in comparison with ANC follow up

**Fig 2:**
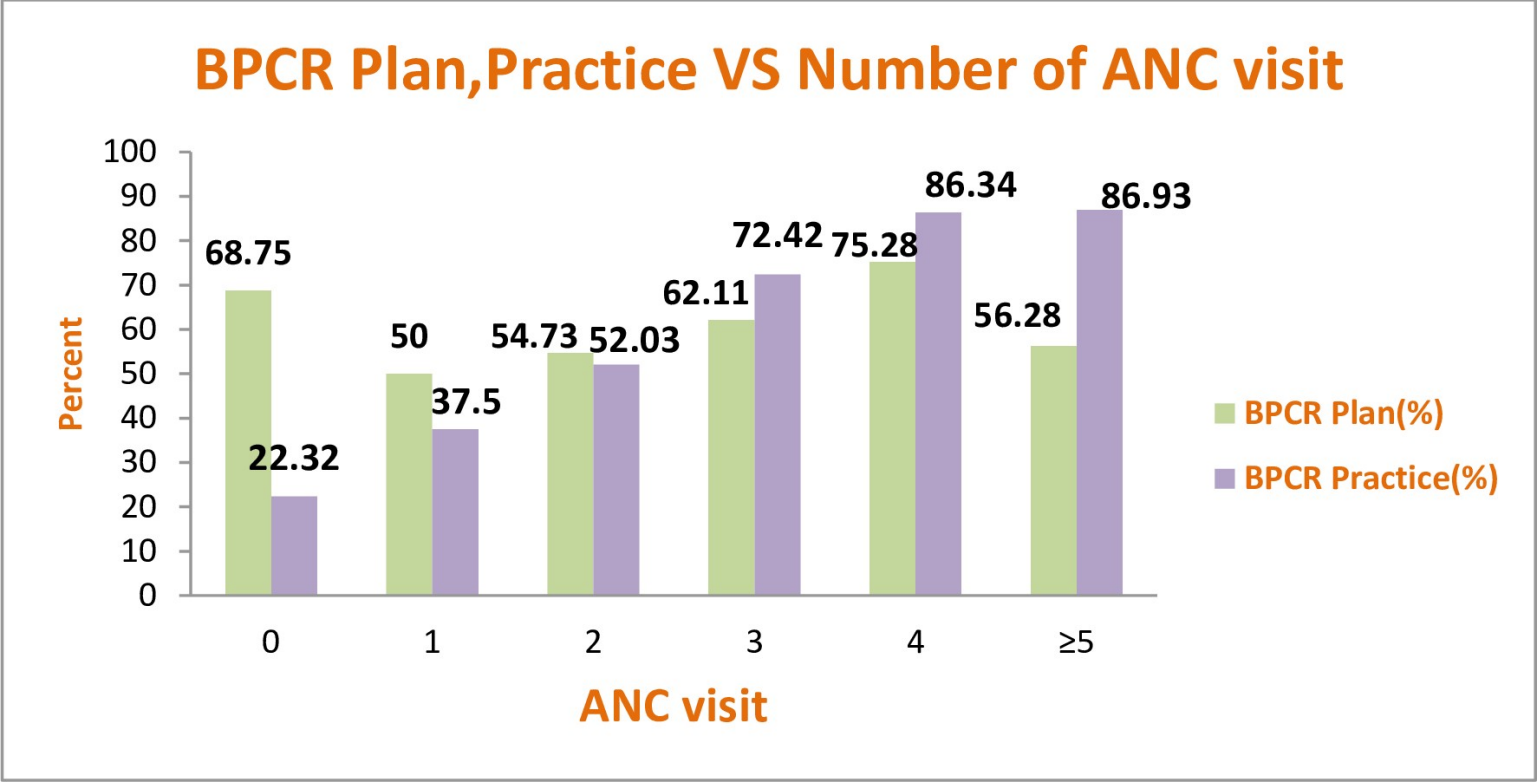
BPCR plan and practice versus number of antenatal care visits among pregnant women in North Gondar Zone, Northwest Ethiopia, 2018.

The BPCR practice of the women was higher 86.3% when a woman attended four ANC follow-up. As the frequency of visits increased more than 3 times, both the planning and the actual practice of BPCR was increasing simultaneously. Pregnant women who do not attend ANC follow up, not only have a smaller BPCR planning and practicing but also their actual practice at the time of delivery was lower than what they claimed they planned to do during pregnancy (Fig 2).

### BPCR plan and BPCR practice of the women

From the components of the BPCR, the most practiced at the time of delivery were identifying labor companion (91.73%), preparing essential items for delivery (90.81%), and identifying support person for the household (88.09%). Whereas arranging potential blood donors (20.49%) and identifying skilled attendants (67.54%) were the least practiced elements during delivery.

All of the components of BPCR were practiced than they were planned except for identifying the place of delivery which showed a slight decrement (86% to 85%) in actual practicality of the plan (**Table 3**).

**Table 3:** Individual BPCR plan and practice among the cohort of pregnant women in North Gondar Zone, Northwest Ethiopia, 2018 (n = 1523).

Variable	BPCR Plan Frequency (%)	BPCR Practice Frequency (%)
Identified place of delivery	1314(86.28)	1295(85.03)
Save money	1100(72.23)	1230(80.76)
Identified skilled birth attendant	611(40.12)	1028(67.54)
Identified means of transportation	1128(74.04)	1064(69.86)
Identified place of health institution to give birth	1217(79.91)	1158(76.03)
Arranged potential blood donor	255(16.74)	312(20.49)
Identified labor companion	1230(80.76)	1397(91.73)
Prepared essential items for labor and delivery	888(58.31)	1383(90.81)
Identified support person for the household	991(65.07)	1339(88.09)

### Factors associated with BPCR

On bivariate analysis, factors like, maternal age, education, husband education, residence, number of families, types of health facility, Wealth index, number of pregnancies, planning of pregnancy, Decision on maternal health care utilization, knowledge of danger signs of pregnancy, time ANC is initiated in months, number of ANC visits counseling on BPCR and counseling on institutional delivery were significantly associated with BPCR at p-value <0.2.

Variables which have significant association in multivariate analysis were; Number of ANC visit [AOR: 1.97 (95% CI:(1.67–2.32)], number of family in the household [AOR: 1.14 (95%CI:(1.00–1.30)], highest wealth index [AOR:1.87(95%CI:(1.16–3.01)], Multigravidity [AOR: 0.30 (95% CI: (0.15–0.62)] joint decision making with a spouse [AOR:2.2 (95% CI: 1.25, 3.82)], get counseling about BPCR [AOR: 2.35 (95% CI:(1.51–3.68)], were found significantly associated with BPCR practice **(Table 4).**

**Table 4:** Bivariate and Multivariate analysis of factors associated with BPCR Practice among pregnant women, North Gondar Zone, Northwest Ethiopia, 2018.

Variable	BPCR practice		COR (95%CI)	AOR (95%CI)
	Yes	No		
**Age**			0.97(0.95–0.99)	1.00(0.96–1.03)
**Number of family in the household**			**0.91(0.86–0.97)**	1.14(1.00–1.30)*
**Number of ANC visit**			**2.06(1.87–2.28)**	1.97 (1.67–2.32)**
**Mothers education**				
unable to read and write	451	225	1	1
able to read and write	113	45	1.25(0.85–1.83)	1.07(0.67–1.70)
primary education	193	59	1.63(1.17–2.27)	1.38(0.87–2.18)
secondary education	205	54	1.89(1.34–2.66)	1.39(0.82–2.36)
college and above	158	20	3.94(2.40–6.44)	1.66(0.79–3.49)
**Husbands education**				
unable to read and write	316	154	1	1
able to read and write	234	85	1.34 (0.97–1.83)	0.94 (0.64–1.40)
primary education	174	75	1.13 (0.81–1.57)	0.64 (0.41–1.00)
secondary education	198	53	1.82 (1.27–2.60)	0.70 (0.41–1.21)
college and above	173	24	3.51 (2.19–5.61)	0.83 (0.40–1.72)
**Residence**				
Rural	458	234	1	1
Urban	662	169	2.00(1.57–2.29)	0.85(0.56–1.28)
**Public transport access**				
No	247	169	1	1
Yes	873	234	2.55(2.00–3.25)	1.25(0.89–2.00)
**Type of health facility**				
Health post only	371	194	1	1
Health center and above	732	200	1.91 (1.51–2.41)	0.92 (0.59–1.42)
**Wealth index**				
Lowest	195	111	1	1
Second	189	128	0.84(0.6–1.16)	0.77 (0.52–1.16)
Middle	**240**	**62**	**2.2(1.53–3.16)**	2.08 (1.34–3.21)**
Fourth	**248**	**53**	**2.66(1.82–3.88)**	2.08 (1.29–3.15)*
Highest	**248**	**49**	**2.88(1.96–4.23)**	1.87 (1.16–3.01)*
**Number of pregnancies**				
1	362	73	1	1
2–4	**532**	**208**	**0.51 (0.38–0.69)**	0.40 (0.26–0.62)**
> = 5	**226**	**122**	**0.37 (0.26–0.52)**	0.30 (0.15–0.62)**
**Is the current pregnancy planned**				
No	104	48	1	1
Yes	1,016	355	1.32(0.91–1.89)	1.07 (0.66–1.73)
**A decision on maternal health care utilization**				
Me alone	59	42	1	1
Me and my husband	**1,003**	**345**	**2.06(1.36–3.13)**	1.92 (1.07–3.44)*
Others	**58**	**16**	**2.58(1.30–5.09)**	2.81 (1.15–6.86)*
**Knowledge of danger signs of pregnancy**				
No	713	288	1	1
Yes	407	115	1.42(1.11–1.83)	1.17 (0.85–1.56)
**Counseled about birth preparedness and complication readiness plan**				
No	872	375	1	1
Yes	248	28	3.80(2.53–5.73)	2.35 (1.51–3.68)**
**ANC utilization**				
No	25	87	1	1
Yes	1,095	316	12.0(7.6–19.1)	0.86(0.40–1.80)
**BPCR plan**				
No	165	350	1	1
Yes	770	238	1.52(1.20–1.92)	1.26(0.94–1.69)
**Counseled on importance of institutional delivery**				
No	539	260	1	1
Yes	581	143	1.95(1.54–2.47)	1.33(0.99–1.79)

Note * = P-value <0.05;

** = p-value <0.001.

## Discussion

BPCR plan is a simple and easily implemented strategic tool that helps a woman to have a safe and sound labor and delivery experience. It can be implemented at any time during pregnancy thereby eases the women’s burden of decision making at the time of labor and delivery. Antenatal care is one of the windows of opportunities to educate the women about BPCR for every woman attending the services. However, sometimes this simple strategy is not implemented appropriately or the women fail to practice what has been implemented at the time of her pregnancy when labor starts. This study assessed the BPCR plan of a woman at the time of pregnancy as well as her actual practice during labor in five Woredas of North Gondar Zone.

The prevalence of BPCR practice at the time of birth was 73.5 [95% CI 71.3–75.7]. This is lower than the study conducted in Karnataka, India (79.3%). The possible explanation for this could be, the Indian study included women who attended Primary Health Care (PHC) services. Whereas in our study we enrolled all pregnant women in the community [21] besides, there is a difference in the socio-economic characteristics of respondents. On the other hand, our finding is higher than studies conducted in Southern Ethiopia 48.5%, Anguak 23.4%, and Goba 29% [18,22,23]. The possible explanation for this difference could be the difference in the study population, and time gap. In Southern Ethiopia and Goba studies, women within 12 months postpartum were included which may lead to recall bias and decreased the prevalence. The study in Agnuak, on the other hand, includes pregnant women starting from the 3^rd^ months of pregnancy which may decrease the prevalence as many women practice BPCR later in pregnancy.

Frequent ANC visits were one of the factors for BPCR practice at the time of birth. For a unit increase in the ANC attendance, there was a 19.8% increase in BPCR practicing. This is in line with studies conducted in Agnuak, chamwino district Tanzania and North West Ethiopia [19,22,24] were BPCR increased as the women attended four and above ANC follow. The possible explanation for this could be, women who attended the recommended number of ANC visits might get BPCR counseling at every visit, which in turn will help her to retain the repeated advice given by the health care professionals. However, even though the number of ANC visits highly determined the BPCR practice of the women, 8.6% of the women who attended the recommended ANC visit failed to be prepared for birth. This might show there is still a gap in the implementation of BPCR among full ANC utilizers.

For a unit increase in the family size, there was an 11.4% increased chance of the BPCR practice. This is supported by a study conducted in Adama town [25] where women were found to be more prepared as the number of families in the household was higher. The possible explanation might be as the number of the family increases it will be easier for the women to assign tasks such as selecting blood donor with compatible blood group, to the people who live near her as BPCR is not solely the responsibility of the women but also her family [15].

Women having the highest wealth assets were 2.01 times more likely to practice BPCR than women in the lowest quartile. This is supported by studies conducted in India [21]. The possible explanation for this might be people in the highest quartile will have the means to save money for emergency funding and transportation.

Mothers who became pregnant for the first time practiced BPCR than those mothers who are pregnant with their second child or more. Mothers who are pregnant for the second time or above were 43% less likely to practice BPCR. This is supported by the studies conducted in Southern Ethiopia and Wolaita Zone [26,27]. The possible explanation for this might be since multigravida mothers have prior experience of having a child it may make them reluctant towards the pregnancy than the primiparous women who have perceived experience of childbirth as a high-risk condition. The findings of our study were contrary to studies conducted in rural India and Adigrat where multigravida women were found to practice BPCR [28,29].

Women who involve their husbands on maternal health care utilization decision making were 1.9 times more likely to practice BPCR than women who decided by themselves. This might be because, in many countries in Africa including Ethiopia where the majority of the breadwinners are males, and men have the economic and decision making power over maternal health care utilization [30] if the husband is involved in the decision-making process, he is more likely to support her with what is needed to execute BPCR practice, including money provision, decision during the time of emergency and accompanying her during the labor and delivery which will enable her to utilize maternal health care services early and prepare for birth and possible complication that may arise. This finding is supported by studies conducted in Kenya and Bangladesh [15,31,32].

Women who were advised on BPCR were 2.35 times more likely to practice during labor and delivery. This is supported by a study conducted in Adigrat Town [29]. The possible explanation for this might be, if a woman is counseled appropriately about BPCR during pregnancy she will be able to implement it at the time of delivery even if they failed to plan ahead of time.

## Conclusion

The prevalence of BPCR practice was higher than studies conducted in other parts of the country. However, BPCR practice was found to be lower than the standard that every woman should practice the plan at the time of delivery. Intersectoral collaborative interventions required to improve the economic status and living standard of families in the community as well as multiple awareness creation strategies should be implemented to support women to attend ANC follow up visits. Moreover community mobilization and women empowerment platforms to promote joint decision making related to maternal health service utilization between partners.

### Strength and limitations of the study

#### Limitations

As the results were based on self-report, there might be social desirability bias which may increase the prevalence.

#### Strength

We tried to assess the BPCR plan of the women during pregnancy as well as the actual practice immediately after delivery; which will significantly reduce the recall bias. Being a community-based study with a larger sample size including urban and rural residents, it could manifest the actual problem at the community level.

## Supporting information

S1 File Raw SPSS dataset. (SAV)

## Abbreviations

ANC: Antenatal Care
BPCR: Birth Preparedness and Complication readiness
EDHS: Ethiopian Demographic and Health Survey
MHC: Maternal Health care
MMR: Maternal Mortality Ratio
SDG: Sustainable Development Goals
WHO: World Health Organization
